# Coarctation of the Aorta as a Complication of Surgical Ligation of Patent Ductus Arteriosus in a Premature Infant

**DOI:** 10.1155/2017/2647353

**Published:** 2017-03-13

**Authors:** Amna Qasim, Soham Dasgupta, Sunil K. Jain, Amyn K. Jiwani, Ashraf M. Aly

**Affiliations:** ^1^Department of Pediatrics, University of Texas Medical Branch, Galveston, TX 77550, USA; ^2^Department of Neonatology, University of Texas Medical Branch, Galveston, TX 77550, USA; ^3^Department of Pediatric Cardiology, University of Texas Medical Branch, Galveston, TX 77550, USA

## Abstract

Surgical ligation of a patent ductus arteriosus (PDA) is a commonly performed procedure. Complications are infrequent and most commonly include recurrent laryngeal nerve injury and rarely ligation of left pulmonary artery. We report a case of accidental ligation of the descending thoracic aorta leading to a clinically significant coarctation.

## 1. Introduction

Surgical ligation of patent ductus arteriosus (PDA) is a commonly performed procedure with infrequent complications such as pneumothorax, tears in the ductus arteriosus or the aorta, wound infections, injury of the recurrent laryngeal nerve, and ligation of the left main bronchus or the left pulmonary artery [1]. We report the first known case of accidental clipping of the descending aorta leading to a clinically significant coarctation during a surgical ligation of a PDA.

## 2. Case Presentation

A premature female infant born at 32 weeks weighing 1,410 grams had a complicated neonatal course that included respiratory distress requiring mechanical ventilation and necrotizing enterocolitis that required exploratory laparotomy and bowel resection. A long systolic murmur was heard at 2 weeks of life. An echocardiogram revealed a large PDA (left to right shunt), mild left atrial and left ventricular dilation, and a normal aortic arch. The baby was treated with 2 courses of indomethacin without any response. A chest X-ray showed cardiomegaly and pulmonary congestion consistent with congestive heart failure. Since the patient failed decongestive therapy, surgical ligation of the PDA was performed on day 18 of life.

**Figure 1:**
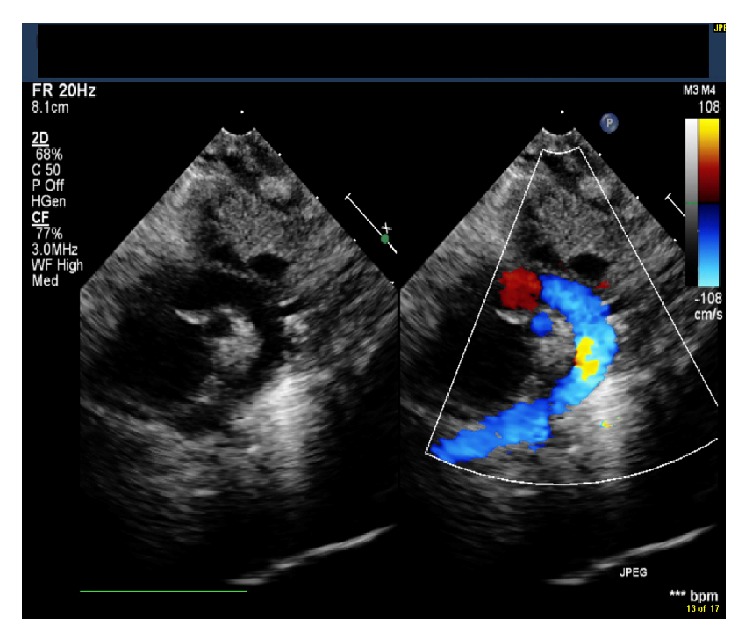
Echocardiogram (suprasternal view) demonstrating a normal aortic arch without any evidence of coarctation prior to patent ductus arteriosus ligation surgery.

**Figure 2:**
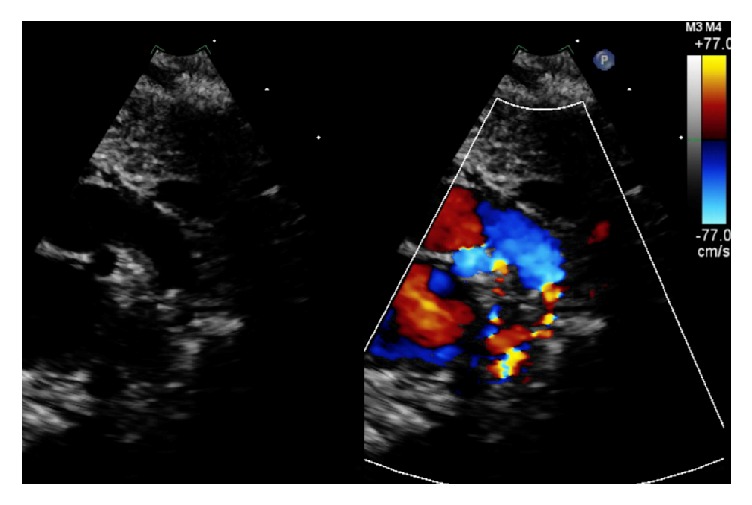
Echocardiogram demonstrating juxtaductal coarctation of the aorta following patent ductus arteriosus ligation surgery.

A left posterolateral approach was used and the aortic arch, descending aorta, left subclavian artery, vagus nerve, recurrent laryngeal nerve, and PDA were all reported to be visualized clearly. The PDA was ligated with a 10 mm clip after ensuring adequate pre- and postductal saturations. The patient was subsequently noted to have a loud ejection systolic murmur. The blood pressure in the upper extremities was noted to be higher than in the lower extremities (91/65 mmHg versus 77/45 mmHg, resp.). A repeat echocardiogram demonstrated juxtaductal coarctation of the aorta due to impingement of the PDA clip on the descending thoracic aorta (Figure 1 versus Figure 2). The peak velocity was 4.1 m/sec and the peak gradient was 68 mmHg. After extensive discussion with the surgical team, it was decided to monitor the patient clinically with the anticipation that she may need surgery if her condition deteriorates. Follow-up echocardiograms demonstrated depression of the left ventricular systolic function (shortening fraction down to 17%) with dilation of both left atrium and ventricle. At that point, surgical intervention was deemed necessary and the coarctation was corrected with a patch repair technique. Postsurgical echocardiograms revealed improvement in cardiac function (shortening fraction 27–29%) and no residual coarctation. The infant is currently hemodynamically stable from a cardiovascular standpoint.

## 3. Discussion

A patent ductus arteriosus is necessary during fetal life. The PDA usually closes spontaneously shortly after birth. The factors that enhance PDA closure include a high oxygen tension and a decrease in endogenous prostaglandins. The incidence of PDA is higher in premature infants. Prostaglandin synthetase inhibitors such as indomethacin are commonly used to induce ductal closure in the first 2–4 weeks of life [2]. Symptomatic patients who fail medical management are likely candidates for surgical ligation which is commonly done at the bedside.

Surgical treatment has been shown to be safe and effective, with only occasional complications. The main surgical complications include recurrent laryngeal nerve injury, need for chest tube placement, diaphragmatic paralysis, and wound infections [3]. There have been rare reports of ligation of the left main bronchus and/or the left pulmonary artery [4] and nicking of the aorta/ductus arteriosus [5]. Over the years, less invasive transcatheter methods for closure of PDA have been developed. Although this is the preferred method in older children, it may not be amenable in small premature infants. Common complications of the transcatheter approach include embolization and vascular injuries. A large meta-analysis showed that both surgical and transcatheter approaches for PDA closure have comparable outcomes. Reintervention is more common with catheter-based interventions, but overall complication rates are similar and hospital stay is significantly shorter [6].

To our knowledge, this is the first reported case of accidental clipping of the aorta leading to clinically significant coarctation during a surgical ligation of a PDA. The surgical ligation involves meticulous dissection to identify the subclavian artery, descending aorta, distal arch, and ductus before ligation. Great care must also be taken to avoid injury to the left recurrent laryngeal nerve. To avoid such injury, surgeons should aim to clip the ductus as far away from the nerve as possible. Coarctation of the aorta may present with hypertension in the upper extremities, headache, lower extremity claudication, and left ventricular hypertrophy. Acute coarctation of the aorta, as seen after accidental clipping, can potentially lead to spinal arachnoid hemorrhage and lower extremity paraplegia and hypoesthesia as reported in adults [7].

## 4. Conclusion

We report a rare case of accidental clipping of the aorta during surgical ligation of PDA in a premature infant. It is essential that the surgeon is aware of this potential complication. Measurement of pre- and postductal saturations and upper and lower extremity blood pressure and performing echocardiography during or immediately after surgery may allow earlier identification of this complication.
